# Association between visual classification of kyphosis and future ADL decline in community-dwelling elderly people: the Kurabuchi study

**DOI:** 10.1007/s11657-018-0551-4

**Published:** 2018-12-18

**Authors:** Keiko Sugai, Takehiro Michikawa, Toru Takebayashi, Morio Matsumoto, Masaya Nakamura, Yuji Nishiwaki

**Affiliations:** 1 Department of Orthopaedic Surgery, Keio University School of Medicine, Tokyo, Japan; 2 Department of Environmental and Occupational Health, School of Medicine, Toho University, 5-21-16 Omorinishi, Ota-ku, Tokyo, 143-8540 Japan; 3 Department of Preventive Medicine and Public Health, Keio University School of Medicine, Tokyo, Japan

**Keywords:** Cohort study, Kyphosis, Activity of daily living, Visual assessment

## Abstract

***Summary*:**

This cohort study conducted in Japan showed that severe age-related kyphosis was visually detected. The visual assessment of kyphosis was associated with declines in ADL, suggesting that we can easily identify people at high risk to develop future ADL reduction in the community setting.

**Purpose:**

Age-related kyphosis is related with declines in activities of daily living (ADL). Its conventional diagnosis has been made by orthopedic surgeons and trained examiners using specialized equipment such as X-rays. We investigated whether visual classification of kyphosis by laypersons accurately predicted future ADL decline.

**Methods:**

This study was part of the Kurabuchi Study, a cohort study of community-dwelling elderly Japanese. Between 2009 and 2010, three layperson raters used reference illustrations to classify 532 participants without ADL decline at study baseline into four categories. Other examiners used conventional methods to assess kyphosis in the same participants: curve ruler, Spinal Mouse, and the block method. ADL decline was defined as the development of dependence according to the Katz Index, admission to a nursing home, or certification of long-term care need.

**Results:**

Thirty-five of the participants (6.6%) were classified with the most severe degree of kyphosis at baseline by visual assessment. Interrater agreement was high (Kappa = 0.73) for the most severe group. During 4.5 years of follow-up, 106 participants (19.9%) showed ADL decline. On the basis of visual assessment, the adjusted risk ratio for ADL decline among the participants with the most severe kyphosis was 2.6 (95% CI: 1.4–4.6). Assessments of kyphosis made with the Spinal Mouse also accurately predicted ADL decline.

**Conclusions:**

Visual assessment of kyphosis predicted future declines in ADL in this study. Since our method requires no special tools or training, it may be useful for identifying those at high risk of ADL decline.

**Electronic supplementary material:**

The online version of this article (10.1007/s11657-018-0551-4) contains supplementary material, which is available to authorized users.

## Introduction

Scientific advances have led to longer lifespans and aging societies. According to United Nations data, more than 40% of the populations of several Asian and European countries will be over the age of 60 by 2050; among these countries, Japan has the highest aging rate [1]. Clearly, it is desirable to keep the elderly members of these aging societies as physically independent as possible by identifying those at risk of developing dependency and taking appropriate measures to prevent it [2].

As the number of healthy elderly people increases, it becomes more difficult to predict functional deterioration only on the basis of age. Therefore, to identify those who would benefit from preventive intervention, effective predictive measures are needed. Various conditions like dementia, depression [3], pain [4], and vision and hearing impairments [5] have been reported as useful predictors of declines in activities of daily living (ADL) in the elderly, and we have previously reported that measurement of age-related kyphosis with a combination of the block method and kyphosis index [6], and with the Spinal Mouse [7] can also predict future ADL decline.

Some 20 to 30% of elderly people reportedly develop age-related kyphosis [8]. Diagnosis has conventionally been made by orthopedic surgeons and trained examiners using such special tools as the curve ruler [9], blocks [10], markers [11], and X-ray images [12], with patients standing upright or lying down. However, severe kyphotic deformities can generally be easily evaluated simply on the basis of appearance, even by laypersons. If simple visual classification of age-related kyphosis by laypersons can accurately predict future ADL declines, it can be applied during community health examinations, which are usually performed by public health nurses without special training in kyphosis assessment.

The purpose of this study was to investigate whether visual classification of kyphosis by laypersons is useful. We hypothesized that this classification method would predict the future ADL decline of community-dwelling elderly.

## Methods

### Study population

This study was performed as part of the Kurabuchi Study [4–7, 13–15], a community-based cohort study that has targeted all residents aged 65 years and older in Kurabuchi Town (Takasaki City, Gunma Prefecture, Japan) since 2005. This mountain town located in the central part of Japan had a population of about 4800 in 2004, and about 25% of the residents are engaged in agriculture or forestry. Excluding those who were hospitalized or institutionalized, 1294 residents were identified in 2005 and 2006 as eligible for participation in the study, and 834 (64.5%) of them took part in the first health survey. This was followed by annual follow-ups.

In 2009 and 2010 (the baseline for the present study), an extra item—classification of kyphosis by visual assessment—was included in the health examinations performed at the eight community centers. Among the residents, 560 participated in this baseline survey, 406 of whom were original participants from the 2005–2006 survey, 81 of whom had newly entered the 65-year-old age bracket, and 73 of whom had newly decided to participate in the study since the first survey. At this point, we excluded 25 participants with ADL decline at baseline: 21 with certification of long-term need of support, 9 with dependence in ADL (Katz ADL) [16], 4 who had been admitted to nursing homes (there were several in more than one category). We further excluded 1 with cerebral palsy, and 1 with kyphosis too severe to assess. Follow-up ADL data was obtained annually until March 2014 (mean follow-up period: 4.5 years). Excluding 1 individual who moved out of the town during the follow-up, 532 participants (242 men, 290 women) were left for the final analysis.

The study protocol was approved by the Ethics Committees of Keio University School of Medicine (Tokyo, Japan) (Approval No.16-20) and Faculty of Medicine, Toho University (Tokyo, Japan) (Approval No. 2700623046), and written informed consent was obtained from all participants.

### Classification of kyphosis by visual assessment

**Fig. 1:**
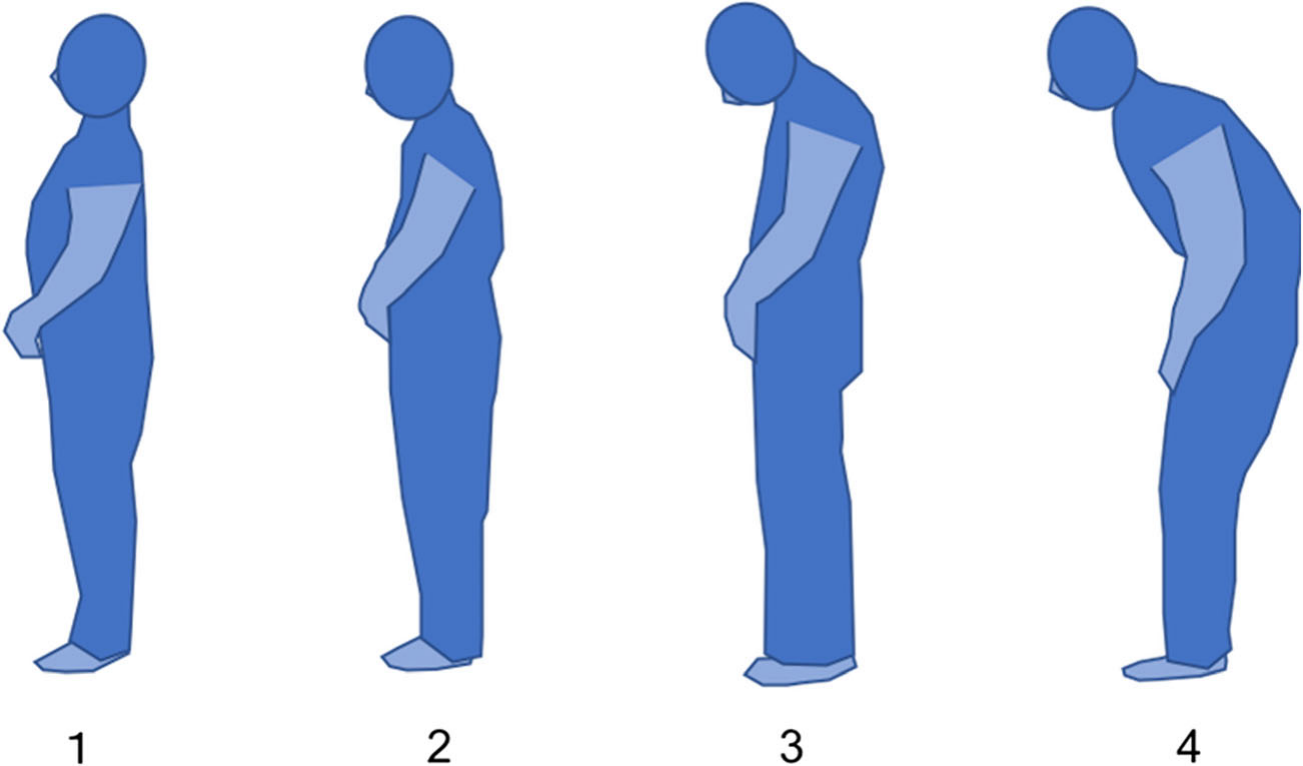
Pictures used for reference in the visual classification of kyphosis (these were drawn from photographs, but the originals were not used so as to protect the privacy of the patients)

For the purposes of this study, we developed a simple evaluation method in which the rater uses reference illustrations (Fig. 1) to visually classify a participant’s degree of kyphosis into 1 of 4 categories: 1, no kyphosis; 2, slight kyphosis; 3, between 2 and 4; or 4, severe kyphosis. The rater does not require the participant to adopt any specific position for the evaluation but simply classifies the degree of kyphosis according to the participant’s posture when he or she is moving around normally. We recruited three raters (A, B, and C), none of whom are orthopedic specialists, and masked each to the classifications made by the other 2, and to the kyphotic evaluation results obtained by conventional methods.

### Measurement of kyphosis by conventional methods

The degree of kyphosis was also assessed by three different conventional methods: the kyphosis index (KI, standing position), Spinal Mouse (standing position), and the block method (supine position). All three types of conventional assessment were performed by trained examiners.

For KI assessments, the participants were asked to relax and stand naturally, and the thoracic curvature between the spinous processes of the seventh cervical and fourth lumber vertebrae was traced onto paper with an adjustable curve ruler; KI was then calculated according to the formula proposed by Milne and Lauder [9]. Since no cutoff value for KI has been established, we classified the results into sex-specific quartiles.

In the second conventional method, the participants were again asked to stand in a relaxed position, and spinal posture was evaluated with a Spinal Mouse (Indiag, Volkerswill, Switzerland), a computer-assisted, noninvasive device to measure spinal shape; measurements were made as described elsewhere [7]. From the available parameters, we selected inclination (the angle between a straight line from Th1 to S1 and true vertical) [17] for this study. Since no cutoff value for inclination has been suggested, we classified the results into sex-specific quartiles, as in our previous research [7].

For the block method, the distance between the participant’s occiput and the table was measured with blocks, each measuring 1.5 cm in height. The blocks were placed under the neck with the participants in the supine position, lying with their face parallel to the floor, and the number of blocks used for each participant was recorded [6, 18]. We showed in a previous study that there is no difference in the risk ratio (RR) for ADL decline with measurements between 0 and 2 blocks [6], so we classified the results into four categories: 0–2 blocks, 3 blocks, 4 blocks, or ≥ 5 blocks.

### Outcome measurements

The participants were followed up annually and assessed for any dependence in ADL. We defined dependence in ADL as previously reported [6]: admission to a nursing home, certification of a need for long-term care/support, or a need for help in any of the six basic ADL items in the Katz Index [16]. We certified a participant as dependent in ADL if he/she met at least one of the three criteria once or more during the follow-up period, and defined this condition as “combined ADL decline.”

Information on nursing home admission, certification of a need for long-term care/support, and death was collected from the Kurabuchi Branch Office of Takasaki City Hall. Information relevant to Katz ADL classification was collected during the annual home-visit surveys.

### Covariates at baseline

Information was collected during the baseline survey in 2009 and 2010 on age, sex, current smoking status (yes/no), current drinking status (yes/no), living status (with spouse/family or alone), history of life-threatening diseases (stroke, coronary heart disease, diabetes mellitus, cancer—summary answer of yes or no), current knee joint pain (always, often, occasionally, or never), current back pain (always, often, occasionally, or never). The demi-span [13] was used to estimate body mass index (BMI), and participants were categorized as underweight (< 18.5), normal weight (18.5–24.9), or overweight (≤ 25). Information on marital status (married vs widowed/divorced/single) and educational level (high school or higher vs junior high school or lower) was obtained from the first survey in 2005 to 2006. Because vision impairment, hearing handicap, and depressive symptoms are also known to be associated with ADL decline [5, 19–21], we also included these variables as potential confounders. Vision impairment was defined as a corrected distance visual acuity of worse than 0.5 in the better eye, as measured with a Landolt broken ring chart at 5 m, according to the US criteria [22]. Hearing handicap was defined as a score of 10 or more on the 10-item screening version of the Hearing Handicap Inventory for the Elderly (HHIE-S) [23]. Depressive symptoms were defined as a score of 2 or more on the Geriatric Depression Scale 5-item version (GDS5) [24]. Bone stiffness, which is thought to be related to kyphosis, was measured in the calcaneus with a Q-1000 Express (GE Yokogawa Medical Systems, Tokyo, Japan) using a quantitative ultrasound bone mass measurement system, and the results were classified into sex-specific quartiles.

### Statistical analysis

STATA version 14 (STATA Corp., College Station, TX) was used for all data analyses.

The participants were divided into four groups according to the visual classification of kyphosis by each rater. The Chi-square test or Fisher’s exact test was used to compare the baseline variables among the four kyphotic classifications. The observer agreement between the raters was assessed using Fleiss’s Kappa [25].

The association of each kyphosis category, as classified visually, with ADL decline, was assessed by crude analysis, and then by three multivariate analyses using Poisson regression models, with category 2 (into which the largest number of participants fell) used as the reference. The first model was adjusted for age categories and sex. Model 1 included marital status, education, drinking, smoking, BMI, vision impairment, hearing handicap, knee joint pain, depressive symptoms, and history of life-threatening diseases, which were considered a priori confounders or were associated with visual kyphotic classification, as well as age categories and sex. In addition to the variables adjusted for in Model 1, back pain and bone stiffness (categories) were added in Model 2. The strengths of associations were indicated by the RRs and 95% confidence intervals (CIs).

Finally, the association of each conventional kyphotic measure (KI, inclination, and number of blocks) with ADL decline was assessed by crude analyses, and then by the same three multivariate analyses, with Poisson regression models used for the visual classification. In assessing KI and inclination, the second quantile was used as the reference. For the block method, the 0–2 block category was used as the reference.

## Results

Rater A (the representative rater) rated all 532 participants, rater B assessed 485, and rater C 396. Rater A put 98 (18.4%) of the participants in category 1 (no kyphosis), 294 (55.3%) in category 2 (slight kyphosis), 105 (19.7%) in category 3 (between 2 and 4), and 35 (6.6%) in category 4 (severe kyphosis). Observer agreement between the three raters showed a high rate of agreement (Kappa = 0.73) for category 4, and moderate agreement across all four categories (Kappa = 0.36) (Supplemental Table 1).

The baseline characteristics of the participants in each of the 4 kyphosis categories the representative rater assigned them to are shown in Table 1. The distributions of age, sex, BMI, drinking status, hearing handicap, vision impairment, education, marital status, knee joint pain, back pain, and current depressive symptoms differed across the categories of kyphosis determined by visual classification.

**Table 1:** Baseline characteristics of the participants in the four kyphosis groups as assessed by the representative rater (*n* = 532)

	Kyphosis category								
	1		2		3		4		
	*n* = 98		*n* = 294		*n* = 105		*n* = 35		
	n* (%)								*P* value^#^
Age category									
65–69 years	54	(55.1)	47	(16.0)	4	(3.8)	0	(0.0)	
70–74 years	29	(29.6)	75	(25.5)	18	(17.1)	0	(0.0)	
75–79 years	12	(12.2)	107	(36.4)	24	(22.9)	7	(20.0)	
80 years	3	(3.1)	65	(22.1)	59	(56.1)	28	(80.0)	< 0.001
Sex									
Female	64	(65.3)	135	(45.9)	65	(61.9)	26	(74.3)	< 0.001
Body mass index (BMI)^†^	22.8 ± 3.4		22.9 ± 3.0		22.5 ± 3.3		21.6 ± 3.4		
BMI < 18.5	4	(8.7)	8	(3.7)	10	(11.0)	6	(18.8)	
18.5 =< BMI < 25.0	31	(67.4)	133	(61.9)	59	(64.8)	21	(65.6)	
25.0 =< BMI	11	(23.9)	74	(34.4)	22	(24.2)	5	(15.6)	0.010
Current smoking									
Yes	9	(9.2)	35	(11.9)	10	(9.5)	2	(5.7)	0.625
Current drinking									
Yes	36	(36.7)	118	(40.1)	30	(28.6)	3	(8.6)	0.001
Hearing handicap^⁑^									
Yes	13	(13.3)	82	(27.9)	38	(36.2)	15	(42.9)	< 0.001
Measured vision impairment^§^									
Impaired	89	(90.8)	252	(85.7)	74	(70.5)	24	(68.6)	< 0.001
Education**									
High school or higher	26	(45.6)	71	(28.9)	20	(20.8)	4	(12.5)	0.002
Marital status**									
Married^$^	48	(85.7)	189	(76.5)	75	(78.9)	16	(51.6)	0.003
History of life-threatening diseases^∥^									
Yes	74	(75.5)	203	(69.1)	82	(78.1)	25	(71.4)	0.282
Knee joint pain									
Always	1	(1.0)	26	(8.8)	15	(14.3)	5	(14.3)	
Often	14	(14.3)	30	(10.2)	16	(15.2)	3	(8.6)	
Occasionally	25	(25.5)	89	(30.3)	26	(24.8)	7	(20.0)	
Never	58	(59.2)	149	(50.7)	48	(45.7)	20	(57.1)	0.035
Back pain									
Always	7	(7.1)	35	(11.9)	24	(22.9)	14	(40.0)	
Often	10	(10.2)	28	(9.5)	21	(20.0)	4	(11.4)	
Occasionally	27	(27.6)	94	(32.0)	35	(33.3)	8	(22.9)	
Never	54	(55.1)	137	(46.6)	25	(23.8)	9	(25.7)	< 0.001
Current depressive symptom^¶^									
GDS5 0–1	72	(73.5)	174	(59.2)	48	(45.7)	10	(28.6)	
GDS5 >= 2	26	(26.5)	120	(40.8)	57	(54.3)	25	(71.4)	< 0.001
Bone stiffness									
Q1	8	(8.2)	59	(20.1)	37	(35.2)	23	(65.7)	
Q2	17	(17.3)	83	(28.2)	29	(27.6)	6	(17.1)	
Q3	24	(24.5)	80	(27.2)	21	(20.0)	4	(11.4)	
Q4	49	(50.0)	72	(24.5)	18	(17.1)	2	(5.7)	< 0.001
Occiput to table distance									
0–2	96	(98.0)	239	(81.3)	49	(47.1)	17	(48.6)	
3	2	(2.0)	36	(12.2)	29	(28.9)	3	(8.6)	
4	0	(0.0)	16	(5.4)	15	(14.4)	2	(5.7)	
5-maximum	0	(0.0)	3	(1.0)	11	(10.6)	13	(37.1)	< 0.001
Kyphosis index									
Q1	42	(42.9)	74	(25.1)	14	(13.3)	1	(2.9)	
Q2	33	(33.7)	85	(28.9)	13	(12.4)	2	(5.7)	
Q3	17	(17.4)	76	(25.9)	28	(26.7)	7	(20.0)	
Q4	6	(6.1)	59	(20.1)	50	(47.6)	25	(71.4)	< 0.001
Inclination measured by Spinal Mouse									
Q1	45	(45.9)	60	(20.4)	10	(9.5)	1	(2.8)	
Q2	32	(32.7)	95	(32.3)	12	(11.4)	0	(0.0)	
Q3	20	(20.4)	90	(30.6)	20	(19.5)	3	(8.6)	
Q4	1	(1.0)	49	(16.7)	63	(60.0)	31	(88.6)	< 0.001

*Due to some missing values, the totals for the stratified subgroups are not equal. ^†^Body mass index was calculated as weight (kg) divided by the square of height (m), as predicted by the demispan. ^⁑^Hearing handicap Inventory for the Elderly Screening scores of 10 or more were considered to indicate hearing handicap. Corrected distance visual acuity worse than 0.5 in the better eye. **Answered in the questionnaire used in 2005–2006. ^∥^Past or current history of major illness: stroke, coronary heart disease, diabetes mellitus, cancer. ^¶^Depressive symptoms were defined as scores of 2 or more on the five-item version of the Geriatric Depression Scale. ^#^Chi-square test or Fisher’s exact test. ^$^Widowed, divorced, or single persons are not recorded as Married

During the follow-up, 106 (19.9%) participants showed ADL decline (85 with certification of need of long-term care/support, 63 with low Katz ADL scores, and 21 who had entered nursing homes or hospitals; the figures include overlaps), and 47 (8.8%) died. The adjusted RRs of ADL decline or death according to the representative rater’s kyphotic classifications are shown in Table 2. Category 4 was most strongly associated with each of the three criteria for ADL decline, and with combined ADL decline. However, no association was found between death and visual kyphotic classification, even in the category 4 participants. Similar results were obtained when the visual assessments made by the two other raters were analyzed (Supplemental Tables 2 and 3). For the participants classified as category 4 by the representative rater, the Model 2 results showed an adjusted RR (95% CI) of Katz ADL decline of 3.8 (1.7–8.7), the adjusted RR (95% CI) of nursing home admission was 4.8 (1.2–19.2), and that of certification of need for home assistance was 3.2 (1.6–6.2). The corresponding figures for a combination of these criteria and death were 2.6 (1.4–4.6) and 1.0 (0.1–3.9), respectively.

**Table 2:** Adjusted risk ratios of ADL decline by kyphosis category as assessed by the representative rater

Outcome	Kyphosis category	Outcome		Age and sex adjusted RR^†^	95% CI^⁑^	Model 1^§^ RR^†^	95% CI^⁑^	Model 2^∥^ RR ^†^	95% CI^⁑^
		*n*/*n*	%						
Combined ADL decline*	1	7/93	7.5	0.7	0.3–1.5	0.9	0.4–2.5	1.0	0.4–2.7
	2	44/270	16.3	1.0	–	1.0	–	1.0	–
	3	35/92	38.0	1.9	1.3–2.9	2.1	1.3–3.2	1.9	1.2–2.9
	4	20/30	66.7	3.0	1.9–4.7	3.1	1.8–5.3	2.6	1.4–4.6
Katz ADL decline*	1	2/93	2.2	0.4	0.1–1.6	0.5	0.1–4.0	0.5	0.1–4.1
	2	24/270	8.9	1.0	–	1.0	–	1.0	–
	3	21/92	22.8	2.1	1.2–3.7	2.5	1.3–4.6	2.2	1.2–4.1
	4	16/30	53.3	4.4	2.4–8.1	4.6	2.1–9.8	3.8	1.7–8.7
Admission to nursing home*	1	1/93	1.1	0.4	0.0–3.1	0.0	-^#^	0.0	-^#^
	2	7/270	2.6	1.0	–	1.0	–	1.0	–
	3	9/92	9.8	3.1	1.2–7.7	3.8	1.2–11.7	4.8	
	4	4/30	13.3	3.6	1.1–11.7	4.1	1.5–11.7	4.8	
Need of assistance at home*	1	6/93	6.5	0.8	0.3–2.0	1.2	0.4–3.2	1.2	0.5–3.3
	2	32/270	11.9	1.0	–	1.0	–	1.0	–
	3	29/92	31.5	2.1	1.4–3.4	2.3	1.4–4.0	2.2	1.3–3.7
	4	18/30	60.0	3.6	2.2–6.0	3.7	2.0–6.7	3.2	1.6–6.2
Death	1	5/98	5.1	1.0	0.4–2.8	1.0	0.2–4.6	1.2	0.3–5.4
	2	24/294	8.2	1.0	–	1.0	–	1.0	–
	3	13/105	12.4	1.2	0.6–2.3	1.2	0.5–2.7	1.4	0.6–3.2
	4	5/35	14.3	1.2	0.4–3.2	0.9	0.2–3.3	1.0	0.1–3.9

*Participants who died during the follow-up period (*n* = 47) were excluded from the analysis. ^†^*RR* risk ratio. ^⁑^*CI* confidence interval. ^§^Adjusted for age category, sex, marital status, education, drinking, smoking, BMI, vision impairment, hearing handicap, knee joint pain, depressive symptoms, and history of life-threatening diseases (stroke, coronary heart disease, diabetes mellitus, cancer). ^∥^In addition to the variables adjusted in Model 1, back pain, and bone stiffness category are included. ^#^Due to a lack of outcome data, statistical computation was impossible

Table 3 shows the adjusted RRs of combined ADL decline, as assessed by the three conventional methods of evaluating kyphosis. Inclination as measured with the Spinal Mouse showed a clear association with ADL decline, with an adjusted RR (Model 2) of 2.3 (1.2–4.5) in the group showing the most severe inclination (Q4). On the other hand, there were no obvious associations between KI or number of blocks with combined ADL decline.

**Table 3:** Adjusted risk ratios of “Combined ADL decline” as assessed with a conventional method of kyphosis assessment

Kyphotic measures		Combined ADL decline*		Age and sex adjusted RR^†^	95% CI^⁑^	Model 1^§^ RR^†^	95% CI^⁑^	Model 2^∥^ RR ^†^	95% CI^⁑^
		*n*/*n*	%						
Kyphosis index	Q1	27/123	22.0	1.4	0.8–2.3	1.4	0.7–2.6	1.3	0.7–2.5
	Q2	19/111	17.1	1.0	–	1.0	–	1.0	–
	Q3	20/129	15.5	1.0	0.6–1.8	0.9	0.4–1.7	0.8	0.4–1.7
	Q4	40/122	32.8	1.7	1.0–2.7	1.6	0.9–2.8	1.5	0.8–2.7
Inclination	Q1	14/112	12.5	1.7	0.8–3.5	1.1	0.4–2.7	1.2	0.5–2.9
	Q2	11/127	8.7	1.0	–	1.0	–	1.0	–
	Q3	26/120	21.7	2.4	1.2–4.5	2.1	1.1–4.2	2.1	1.0–4.1
	Q4	55/126	43.7	3.7	2.0–6.8	2.6	1.3–5.0	2.3	1.2–4.5
Number of blocks	0–2	76/373	20.4	1.0	–	1.0	–	1.0	–
	3	12/59	20.3	0.9	0.5–1.5	1.0	0.6–1.9	0.9	0.5–1.6
	4	8/29	27.6	1.0	0.6–2.0	1.5	0.7–3.0	1.4	0.8–2.5
	5 >=	10/23	43.5	1.2	0.8–2.0	0.9	0.5–1.6	1.0	0.5–1.7

*Participants who died during the follow-up period (*n* = 47) were excluded from the analysis. ^†^RR: risk ratio. ^⁑^*CI* confidence interval. ^§^Adjusted for age category, sex, marital status, education, drinking, smoking, BMI, vision impairment, hearing handicap, knee joint pain, depressive symptoms, and history of life-threatening diseases (stroke, coronary heart disease, diabetes mellitus, cancer). ^∥^In addition to the variables adjusted in Model 1, back pain and bone stiffness category are included

## Discussion

Our simple method of having untrained raters use reference pictures to visually classify kyphosis and allowed us to show an association between kyphosis and future ADL decline. There was good agreement among the raters in classifying participants in the most severe category of kyphosis.

Previous studies, most of which were cross-sectional, used various methods to assess kyphosis and also showed an association between kyphosis and ADL decline. For example, Ryan et al. [26] used both qualitative and quantitative methods (visual judgment/apex of the C7 spinous process to wall distance) performed by trained therapists to demonstrate the role of kyphosis in diminished performance on mobility tasks. Tominaga [27] and Yanagida [28] showed an association between occiput-to-wall distance (Wall-Occiput Test) with falls and fear of falls. Takahashi et al. and his team of orthopedic specialists [11] used surface markers to classify subjects into five trunk posture groups while the subjects were standing still in a relaxed position, and reported an association between kyphotic posture and reduced independence in daily outdoor activities.

Our results were consistent with those of studies using conventional methods to classify kyphosis, which generally show the most significant levels of ADL decline in subjects falling into the most severe kyphotic group [6, 10, 26, 27]. In combination, these results strongly suggest that elderly people with severe age-related kyphosis are either currently experiencing ADL decline, or will soon do so. Therefore, screening for severe kyphosis is important not only so that appropriate support can be provided, but also so that future ADL decline can be prevented.

With our visual classification method, 6.6% of the participants were classified in category 4, the most severe kyphosis group, which places them among the 20 to 30% of the elderly population reportedly affected by hyper-kyphosis due to aging [8]; this is the group with the highest risk of developing ADL decline. When it comes to screening to detect those at high risk of ADL decline, simple and cost-effective methods are the most efficacious. To the best of our knowledge, our method of visual classification of kyphosis is the simplest ever reported, since it requires no special training to match people’s kyphotic posture to sample pictures; in addition, our method can be performed anywhere at any time, and it costs almost nothing, making it a very practical way to screen priority subjects as part of community care-prevention services. On the other hand, it should also be noted that our visual assessment does not have higher reliability compared to other methods of posture assessment, such as the Spinal Mouse, the Debrunner kyphometer, the flexicurve, the arcometer, and the inclinometer [29–31]. These alternative methods should be taken when close examination of kyphosis is needed.

Another notable feature of our method is that it allows the subjects to be evaluated while they are moving around normally, meaning that evaluations are based on the posture they adopt in their daily activities. When subjects are asked to stop and stand up straight, their efforts to maintain the position are likely to result in their adopting a posture that is different from their normal one, resulting in an incorrect assessment of kyphosis severity and risk of ADL decline. In addition, in assessing subjects while they are in motion, our visual classification method may be capable of evaluating not only spinal kyphosis, but also muscle strength and the degree of inclination. Inclination in sagittal posture is also reported to be associated with future dependence in ADL [7], and it can be objectively measured with the Spinal Mouse or X-rays. But since these objective methods need to be performed with subjects standing still, they may catch only the moment when the subject stretches his or her posture for measurement. It has been reported that back muscle strength [32] and lower trunk lean mass [33] are associated with kyphosis, and also with inclination and increased risk of the necessity of nursing care [34].

Our study showed no association between the degree of kyphosis and death, although such an association has been reported [18]. It is reasonable to assume that ADL decline can result in early death, and that focusing on kyphosis in the elderly is important from the viewpoint of both precluding long-term care and extending healthy life spans. However, few studies have been carried out on the rehabilitation of elderly people with kyphosis, and its effectiveness in preventing ADL decline is not yet clear [8, 34, 35]. More research into preventive methods and remedies for kyphosis is required.

The major strength of our study is that the participants were community-dwelling elderly people and not patients, which makes the results applicable to the general public. It should also be noted that our follow-up rate was very high. On the other hand, there are three limitations. One is that we lacked information on whether any of the participants received intervention for ADL decline during the follow-up period, and we acknowledge that some may have maintained their ADL levels through effective intervention. Another limitation is that our results could to some extent be Japan-specific: the evaluation criteria for admission to nursing homes or certification of the need of assistance at home, for example, could vary from country to country, and the third limitation is that our visual classification of kyphosis was performed by non-experienced people in our research. Actually, we think it is a good point of our research that our method can be performed at anywhere, and by any person, although, misclassification of kyphosis might reduce if the classification was performed by experienced people such as orthopedic surgeons.

Japan is now experiencing a “super-aging society,” so maintaining the health of its elderly is becoming more and more important, as is the need to involve the community in preventing health impairment in people who are ever more vulnerable to it as they age. Identifying people at high risk is the first step in prevention, and our simple visual method of kyphosis classification can play a role in this. The next step is research to develop interventions that will be effective in helping elderly people with kyphosis maintain good levels of ADL. There are cases that treatable diseases such as Parkinson’s disease, degenerative diseases of lower limbs, or lumber canal stenosis, which accompanies postural change, underlie the condition of kyphosis. In these cases, we should diagnose them sooner in order to make the treatment outcome better. Also, when muscle weakness underlies the condition of kyphosis, approaches to strengthen back extensor muscles may help improve the development of progression of kyphosis, and also improving their QOL [36–39]. However, precise training protocols are still controversial [40], and further study is needed.

## Electronic supplementary material

ESM 1 (PDF 29 kb)

ESM 2 (PDF 49 kb)

ESM 3 (PDF 50 kb)
