# Supplementary material for: Association between visual classification of kyphosis and future ADL decline in community-dwelling elderly people: the Kurabuchi study
Source: Arch Osteoporos. 2018 Dec 18;14(1):3. doi: 10.1007/s11657-018-0551-4 (PMC6299049; doi:10.1007/s11657-018-0551-4)
Supplement: Supplementary file 1 — (PDF 29 kb) [file 11657_2018_551_MOESM1_ESM.pdf]

Supplemental Table 1 Observer agreements on visual assessment of kyphosis among raters A, B, and C

Kyphosis Category	Kappa
1	0.41
2	0.28
3	0.29
4	0.76
combined	0.36
