# Supplementary material for: Association between visual classification of kyphosis and future ADL decline in community-dwelling elderly people: the Kurabuchi study
Source: Arch Osteoporos. 2018 Dec 18;14(1):3. doi: 10.1007/s11657-018-0551-4 (PMC6299049; doi:10.1007/s11657-018-0551-4)
Supplement: Supplementary file 2 — (PDF 49 kb) [file 11657_2018_551_MOESM2_ESM.pdf]

Supplemental Table 2 Adjusted risk ratios of ADL decline by kyphosis category as assessed by rater B

Outcome	Kyphosis Category	Outcome		Age and sex adjusted RR[†]	95% CI[‡]	Model 1[§] RR[†]	95% CI[‡]	Model 2[‖] RR[†]	95% CI[‡]
		n/n	%						
Combined ADL Decline[*]	1	10/102	9.8	0.8	0.4-1.5	0.9	0.4-2.0	1.0	0.4-2.1
	2	51/285	17.9	1.0	—	1.0	—	1.0	—
	3	14/35	40.0	1.8	1.1-3.0	1.8	1.1-3.1	1.5	0.9-2.6
	4	13/21	61.9	2.3	1.4-3.6	2.2	1.3-3.9	2.0	1.1-3.4
Katz ADL decline[*]	1	3/102	2.9	0.4	0.1-1.4	0.4	0.1-1.7	0.4	0.1-1.9
	2	29/285	10.2	1.0	—	1.0	—	1.0	—
	3	10/35	28.6	2.3	1.2-4.4	2.1	1.0-4.6	1.8	0.8-4.0
	4	9/21	42.9	2.7	1.3-5.4	2.9	1.3-6.7	2.5	1.2-5.6
Admission to Nursing Home[*]	1	3/102	2.9	1.6	0.5-5.6	0.5	0.1-5.2	0.6	0.1-5.3
	2	7/285	2.5	1.0	—	1.0	—	1.0	—
	3	3/35	8.6	2.7	0.8-9.7	2.6	0.5-12.3	3.1	0.5-19.1
	4	3/21	14.3	3.1	0.9-10.9	3.3	0.9-12.0	3.8	0.7-21.1
Need of assistance at home[*]	1	9/102	8.8	1.0	0.5-1.9	1.2	0.5-2.7	1.3	0.6-2.9
	2	39/285	13.7	1.0	—	1.0	—	1.0	—
	3	11/35	31.4	1.8	1.0-3.3	1.9	1.1-3.5	1.6	0.9-3.0
	4	13/21	61.9	2.9	1.8-4.8	2.8	1.6-5.0	2.4	1.3-4.4
Death	1	4/106	3.8	0.5	0.2-1.4	0.7	0.3-1.9	0.9	0.3-2.8
	2	32/317	10.1	1.0	—	1.0	—	1.0	—
	3	4/39	10.3	0.9	0.3-2.4	1.0	0.3-3.6	1.1	0.3-4.1
	4	2/23	8.7	0.6	0.1-2.3	0.4	0.1-2.6	0.5	0.1-3.8

Notes:

[*]Participants who died during the follow-up period (n=42) were excluded from the analysis.

[†]RR: risk ratio.

[‡] CI: confidence interval.

[§]Adjusted for age category, sex, marital status, education, drinking, smoking, BMI, vision impairment, hearing handicap, knee joint pain, depressive symptoms, and history of life-threatening diseases (stroke, coronary heart disease, diabetes mellitus, cancer).

[‖]In addition to the variables adjusted for in Model 1, back pain and bone stiffness categories are included.
