# Supplementary material for: Association between visual classification of kyphosis and future ADL decline in community-dwelling elderly people: the Kurabuchi study
Source: Arch Osteoporos. 2018 Dec 18;14(1):3. doi: 10.1007/s11657-018-0551-4 (PMC6299049; doi:10.1007/s11657-018-0551-4)
Supplement: Supplementary file 3 — (PDF 50 kb) [file 11657_2018_551_MOESM3_ESM.pdf]

Supplemental Table 3 Adjusted risk ratios of ADL decline by kyphosis category as assessed by rater C

Outcome	Kyphosis Category	Outcome n/n	Outcome %	Age and sex adjusted RR[†]	95% CI[‡]	Model 1[§] RR[†]	95% CI[‡]	Model 2[‖] RR[†]	95% CI[‡]
Combined ADL Decline[*]	1	11/106	10.4	0.7	0.4-1.3	0.7	0.3-1.6	0.8	0.3-1.7
	2	38/183	20.8	1.0	—	1.0	—	1.0	—
	3	16/48	33.3	1.6	0.9-2.7	1.8	1.0-3.3	1.6	0.8-3.0
	4	12/23	52.1	1.9	1.1-3.3	2.1	1.1-4.2	1.8	0.9-3.6
Katz ADL decline[*]	1	6/106	5.7	0.7	0.3-1.6	0.6	0.2-1.7	0.6	0.2-1.8
	2	23/183	12.6	1.0	—	1.0	—	1.0	—
	3	11/48	22.9	1.8	0.9-3.5	1.8	0.8-4.3	1.5	0.6-3.7
	4	7/23	30.4	1.8	0.9-4.0	1.8	0.6-5.1	1.5	0.5-4.3
Admission to Nursing Home[*]	1	3/106	2.8	0.8	0.3-2.4	0.0	—[#]	0.0	—[#]
	2	8/183	4.4	1.0	—	1.0	—	1.0	—
	3	3/48	6.3	1.6	0.4-6.6	2.8	0.5-15.9	3.0	0.5-16.5
	4	3/23	13.0	2.3	0.6-8.9	4.3	0.9-20.9	3.9	0.4-34.0
Need of assistance at home[*]	1	9/106	8.5	0.9	0.5-1.9	1.0	0.4-2.3	1.0	0.4-2.5
	2	25/183	12.7	1.0	—	1.0	—	1.0	—
	3	13/48	28.1	1.9	1.0-3.7	1.9	0.9-3.8	1.7	0.8-3.5
	4	12/23	52.1	2.8	1.4-5.3	2.9	1.3-5.9	2.4	1.1-5.1
Death	1	3/109	2.8	0.4	0.1-1.6	0.5	0.1-2.3	0.5	0.1-2.2
	2	21/204	10.3	1.0	—	1.0	—	1.0	—
	3	8/56	14.3	1.3	0.6-2.8	1.4	0.6-3.7	1.4	0.5-3.8
	4	4/27	14.8	1.0	0.3-2.7	1.7	0.5-5.6	1.5	0.5-5.1

Notes:

[*]Participants who died during the follow-up period (n=36) were excluded from the analysis.

[†]RR: risk ratio.

[‡] CI: confidence interval.

[§]Adjusted for age category, sex, marital status, education, drinking, smoking, BMI, vision impairment, hearing handicap, knee joint pain, depressive symptoms, and history of life-threatening diseases (stroke, coronary heart disease, diabetes mellitus, cancer).

[‖]In addition to the variables adjusted for in Model 1, back pain and bone stiffness categories are included.

[#]Due to lack of outcome data, statistical computation was impossible.
